# Retrospective evaluation of patients of gastroesophageal reflux disease treated with laparoscopic Nissen's fundoplication

**DOI:** 10.4103/0972-9941.65163

**Published:** 2010

**Authors:** Anish P Nagpal, Harshad Soni, Sanjiv P Haribhakti

**Affiliations:** Department of Surgical Gastroenterolgogy and Advanced Laparoscopic Surgery, Haribhakti Surgical Hospital, Ahmedabad, Gujarat, India

**Keywords:** Laparoscopic Nissen's fundoplication, gastroesphageal reflux disease, outcomes

## Abstract

**AIMS:**

To evaluate retrospectively the outcome of laparoscopic fundoplication in a cohort of patients with typical symptoms of gastroesophageal reflux disease (GERD).

**MATERIALS AND METHODS:**

Forty-two patients with typical symptoms of GERD, who were operated for laparoscopic Nissen's fundoplication from March 2001 to August 2008, were studied. The study was limited to patients with positive findings on upper gastrointestinal (GI) endoscopy done by us and "typical" symptoms (heartburn, regurgitation, and dysphagia) of GERD. Laparoscopic Nissen's fundoplication was performed when clinical assessment suggested adequate oesophageal motility and length. Only one patient who had negative endoscopic findings underwent a 24-h pH monitoring before surgery. Outcome measures included assessment of the relief of the primary symptom responsible for surgery in the early postoperative period; the patient's evaluation of outcome, and quality of life after surgery.

**RESULTS:**

Relief of the primary symptom responsible for surgery was achieved in 95.24% of patients at a mean follow-up of 28 months. Thirty-five patients were asymptomatic, two had minor gastrointestinal symptoms not requiring medical therapy, three patients had gastrointestinal symptoms requiring medical therapy/Proton Pump Inhibitors (PPI) and in two patients the symptoms worsened after surgery. There were no deaths. Clinically significant complications occurred in six patients. Median hospital stay was 3 days, decreasing from 6 days in the first 10 patients to 3 days in the last 10 patients.

**CONCLUSIONS:**

Laparoscopic Nissen's fundoplication is the choice of operation for clinically symptomatic GERD patients.

## INTRODUCTION

The development of laparoscopic fundoplication (LF) over the past several years has resulted in renewed interest in the surgical treatment of gastroesophageal reflux disease (GERD). Historically, effective treatment options for GERD have included life-long antireflux medication and antireflux surgery. LF has been shown to be safe and effective for the treatment of GERD, with 90–94% overall patient satisfaction at long-term follow-up.[1-3] Laparoscopic Nissen fundoplication is positioned to become the standard of surgical care for patients with GERD. Although the advent of LF has increased both patient and physician acceptance of antireflux surgery, most of these procedures are concentrated in centres with demonstrated interest in the surgical treatment of gastroesophageal reflux. Quality of life analyses have become an important part of surgical outcome analysis. Disease-specific questionnaires have been used in an attempt to quantitate the quality of life before and after medical intervention. This study was done to evaluate retrospectively the outcome of LF in a cohort of patients operated by us with typical symptoms of GERD.

## MATERIALS AND METHODS

Forty-two patients with typical symptoms of GERD, who were operated for laparoscopic Nissen's fundoplication from March 2001 to August 2008, were studied. Mean age was 49.7 years and the oldest patient was 78 years. There were 24 (57.14%) male and 18 (42.86%) female patients. The most common symptom was heartburn, followed by regurgitation and constipation. Most patients (97%) were taking proton pump inhibitors for acid suppression and either had breakthrough symptoms or more commonly desired an alternative to lifelong medication. Failure of medical therapy was not required before fundoplication, and all the patients were offered the alternatives of continuing with medical therapy or undergoing antireflux surgery. The study was limited to patients with positive findings on upper gastrointestinal (GI) endoscopy done by us and "typical" symptoms (heartburn, regurgitation, and dysphagia) of GERD. LF was performed when clinical assessment suggested adequate oesophageal motility and length. The indications for surgery were (1) complications of GERD, oesophageal stricture (*n* = 4) and Barrett's oesophagus (*n* = 2); (2) large (>5 cm) sliding or paraesophageal hiatal hernia (*n* = 4); (3) patient's desire to discontinue medical treatment that was controlling reflux oesophagitis (*n* = 32). Out of these 32 patients, hiatus hernia (3–5 cm) was present in 29 patients.

All patients underwent oesophagogastroduodenoscopy prior to surgery. Oesophageal manometry was not done in any patient due to inavailability. Twenty-four-hour oesophageal pH monitoring was performed for only one patient who had negative endosocpic findings but had severe GERD symptoms. All the patients were admitted to the hospital on the day of operation. Antibiotics were not used routinely.

### Operative procedure

The procedure is carried out using general anesthesia with the patient in the lithotomy (or legs apart) position and reverse Trendelenburg position. The surgeon stands in between the legs and the assistant holding the camera stands on the right of the patient and the other assisting surgeon on the left. After placing all trocars, the first step of the procedure is to reduce the herniated stomach from the hiatus. Later we start with the division of the hepatic omentum along the upper lesser curvature of the stomach. This should be done over a short distance to avoid damage to the hepatic branches of the vagus nerve. In one patient, an aberrant left hepatic artery arising from the left gastric artery was discovered and was preserved. A large left hepatic artery arising from the left gastric artery was present in 9.7–18% of patients.[4,5] It should be identified and avoided. In the same patient, aberrant left hepatic vein was encountered which got injured during the dissection in the hepatic omentum near the hiatus on the right side of Gastro Esophageal (GE) junction. The procedure had to be converted into open procedure and later bleeding was controlled.

The next step is to retract the stomach to the left and expose the right crus of the diaphragm. The stomach is retracted to the right-hand side, and the left crus is identified. The oesophagus is elevated, and the posterior vagus nerve can be identified easily behind the oesophagus. Both are lifted and posterior window created. Great care should be exercised in making this opening under clear vision without damaging the posterior wall of the stomach or oesophagus. An umbilical tape is passed posterior to the oesophagus to help in retraction. A complete dissection of the lateral and inferior aspect of the left crus and fundus of the stomach is the key maneuver allowing circumferential mobilisation of the oesophagus.

The left and right crura should be stripped of their surface connective tissue in preparation for the crural closure behind the oesophagus. This usually is accomplished using two to three 2-0 prolene sutures passed through the muscle bundles of the crura. The hiatus is closed, taking care not to snugly tighten it. No bougie is used to close the hiatal opening. The fundus of the stomach is mobilised and about two or three upper short gastric vessels are divided using the ultrasonic scalpel. With caution and meticulous dissection, the fundus can be completely mobilised in almost all patients.

This dissection is done till the fat near the angle of His is reached. The fundus is taken posterior to the lower oesophagus and a loose wrap is made taking care that the anterior wall of the fundus is brought over the anterior wall of the oesophagus above the supporting umbilical tape. The anterior and posterior lips of the fundoplication are sutured together using two or three prolene 2-0 sutures and atleast one suture passing through the anterior oesophageal wall. The wrap created is also fixed to the right crus with one stitch of silk 2-0 or prolene 2-0.

Nissen's fundoplication was performed in all but one patient, in whom the procedure was converted to a Toupet procedure. No patient had previously undergone fundoplication or an oesophageal operation. A diet of clear liquids was begun next day after the operation, and the diet was advanced as tolerated. Individuals were instructed to chew the food well and to eat small meals. Gastrograffin study was not done routinely. Patient was discharged on the second or third postoperative day.

### Follow up

Follow-up time ranged from 1 month to 7 years, with an average of 28 months. Data on operative time, period of hospitalisation, and complications were collected for all patients. Outcome measures included assessment of the relief of the primary symptom responsible for surgery in the early postoperative period, the patient's and the physician's evaluation of outcome, and quality of life evaluation. Out of the 42 patients, 26 were on regular follow up on the out patient basis and the remaining 16 were asked the questionnaire by telephonic conversation. Tables 1‐4 give the questionnaire format.

**Table 1:** Side effects of the operation

Side effect	Number of patients
Temporary swallowing discomfort	1
Dysphagia > 3 months	2
Complaints of bloating	4
Inability to belch	None

**Table 2:** Antireflux medication before and after surgery

	Preoperative	Postoperative
PPI	30	3
H2 blockers	10	-
Antacids	2	-
Calcium channel	-	1
blockers		
Antianxiety drugs	-	1
Prokinetics	-	1
None	-	37

**Table 3:** Patients' assessment of outcome

Status/quality of life evaluation	No. of patients
Cured/excellent	35
Asymptomatic	
Improved/good	2
Relief of primary symptom, but minor GI symptoms, no therapy	
Satisfied with Satisfied with surgery/average	3
Improved, persistent GERD symptoms, therapy required	
Poor/worsened	2
Not improved and/or long-term dysphagia as a consequence of surgical therapy	

**Table 4:** Disease specific questions

Questionnaire	Results
Abdominal pain	None
Heart burn	4
Dysphagia	3
Reflux	3
Constipation	5
Bloating	1

## RESULTS

Relief of the primary symptom responsible for surgery was achieved in 95.24% of patients at a mean follow-up of 28 months. Thirty-five patients were asymptomatic, two had minor gastrointestinal symptoms not requiring medical therapy, and three had gastrointestinal symptoms requiring medical therapy/PPI and in two patients the symptoms worsened [Tables 2 and 3]. Side effects of the operation are summarised in Table 1. Occasional difficulty in swallowing not present before surgery occurred in two patients at 6 months after surgery. Temporary swallowing difficulty was seen in one patient and complaint of bloating sensation in four patients. There were no deaths. Clinically significant complications occurred in six patients. Median hospital stay was 3 days, decreasing from 6 in the first 10 patients to 3 in the last 10 patients.

### Operative complications

In one patient there was bleeding from the left hepatic vein as mentioned above.

### Postoperative complications

One patient had massive pulmonary embolism on fifth postoperative day (POD) but survived later on. Two patients had severe port side infection for which debridement was done in one patient and the other patient had atypical mycobacterium infection treated by AKT. In one patient, Nissen's procedure was converted to Toupet procedure laparoscopically after 2 days, due to severe dysphagia. One patient had left pleural effusion which required ultrasound guided aspiration.

## DISCUSSION

The advent of the laparoscopic approach provides an ideal opportunity to standardise the technique of Nissen fundoplication because it markedly limits the technical variability that can occur with the open procedure. Heartburn is the classic symptom of GERD.[6] Patients with GERD can be divided into those with "typical" symptoms (heartburn, regurgitation, and dysphagia) and those with "atypical" symptoms (cough, hoarseness, and wheezing). Dysphagia is reported by more than 30% of individuals with GERD.[7] Typical symptoms are a more reliable and precise guide to the presence of disease, and consequently their improvement better reflects the effectiveness of therapy. For these reasons, we have chosen to include the disease-specific questionnaire as the basis for the retrospective evaluation of consecutive patients operated for laparoscopic Nissen's fundoplication.

The diagnosis of GERD entails the identification of patients with oesophagitis and its complications as well as patients who have symptoms but no mucosal disease. Endoscopy is mandatory to establish a diagnosis of reflux oesophagitis, to exclude other oesophageal disease and to permit directed biopsy if malignancy is suspected. Measures of oesophageal acid exposure time may be used to quantify reflux before and after treatment; however, if the patient has typical symptoms but no oesophagitis, a temporal association between symptoms and episodes of oesophageal acidification should be sought. Ambulatory 24-hour oesophageal pH-monitoring with accurate event-marking provides recordings suitable for an objective statistical analysis, which was evaluated in only one patient who had typical symptoms of GERD but negative endoscopic findings.

The number of LFs has increased markedly over the last several years. A 10-fold increase in a period of 6–8 years has been reported by several auhors.[9,10] This is mainly due to excellent long-term results, with low morbidity and mortality, as seen in most series of LF performed in patients of all ages.[11,12] Conversion to open laparotomy was necessary in one patient due to injury to left hepatic vein in our series. This injury was associated with aberrant anatomy. An extremely large (12 mm in diameter) aberrant left hepatic vein was probably never encountered before and its proximity to the hiatus made it even more vulnerable. Several authors have reported that the conversion rate may be minimised as the surgeon's experience increases.[9,13]

## CONCLUSION

LF is an effective long-term treatment for GERD and may be performed with a low incidence of postsurgical complications, resulting in high patient satisfaction, improved quality of life, and elimination of antisecretory medicines in the majority of patients.
